# Genetic structure and chemical diversity in natural populations of *Uncaria guianensis* (Aubl.) J.F.Gmel. (Rubiaceae)

**DOI:** 10.1371/journal.pone.0205667

**Published:** 2018-10-26

**Authors:** Isabela Cristina G. Honório, Juliana S. Coppede, Piero G. Delprete, Frederico Henrique S. Costa, Mariana P. C. Telles, Ramilla S. Braga, José Alexandre F. Diniz-Filho, Valéria S. C. Correa, Suzelei C. França, Ana Maria S. Pereira, Bianca Waleria Bertoni

**Affiliations:** 1 Universidade Estadual Paulista “Júlio de Mesquita Filho”, Botucatu, SP, Brazil; 2 Universidade de Ribeirão Preto, Ribeirão Preto, SP, Brazil; 3 Herbier de Guyane, Institut de Recherche pour le Développement, Cayenne, French Guiana; 4 Universidade Federal do Acre, Rio Branco, AC, Brazil; 5 Escola de Ciências Agrárias e Biológicas, Pontifícia Universidade Católica de Goiás, Goiânia, GO, Brazil; 6 Laboratório de Genética e Biodiversidade, Universidade Federal de Goiás, Goiânia, GO, Brazil; 7 Departamento de Ecologia, ICB, Universidade Federal de Goiás, Goiânia, GO, Brazil; 8 Reserva EcoCerrado Brasil, Araxá, MG, Brasil; National Cheng Kung University, TAIWAN

## Abstract

*Uncaria guianensis* is native to the Amazon and is used traditionally as an anti-inflammatory. Natural populations of the species have declined markedly in recent times because of strong anthropic pressure brought about by deforestation and indiscriminate collection. The aim of the present study was to assess the genetic and chemical diversity among eight natural populations of *U*. *guianensis* located in the Brazilian states of Acre, Amapá and Amazonas. A set of four primer combinations was employed in sequence-related amplified polymorphism (SRAP) amplifications of leaf DNA, and the fragments were analyzed in an LI-COR model 4300 DNA Analyzer. Genetic variability within the populations (81%) was substantially greater than that detected between them (19%). The highest percentage of polymorphic loci (90.21%) and the largest genetic variability were observed in the population located in Mazagão, Amapá. Genetic differentiation between populations was high (F_st_ = 0.188) and the studied populations formed three distinct genetic groups (K = 3). The population located in Assis Brasil, Acre, presented the highest average content of the mitraphylline (0.60 mg/g dry weight,). However, mitraphylline and isomitraphylline not detected in most individuals in the studied populations, and it is questionable whether they should be considered as chemical markers of the species. The genetic data confirm the urgent need for conservation programs for *U*. *guianensis*, and for further studies aimed at ascertaining the genetic basis and heritability of alkaloid accumulation.

## Introduction

*Uncaria guianensis* (Aubl.) J.F.Gmel. (Rubiaceae), commonly known as *uña de gato*, is endemic to the Amazonian regions of Bolivia, Brazil, Colombia, Ecuador, Guiana, French Guiana, Peru, Suriname and Venezuela [1]. The plant, which grows as a woody vine that can attain a height of 5–10 m, is used by indigenous populations to treat asthma, arthritis, dermatitis, diabetes, gastritis, inflammation of the genitourinary tract, tumors and ulcers [2]. Pharmacological studies have verified the anticancer, antidiabetic, antimicrobial, anti-inflammatory, antioxidant, anti-Parkinson and immunostimulant effects of the extracts [3]. These properties have been attributed to the presence of pentacyclic oxindole alkaloids (POA), mainly mitraphylline and isomitraphylline, which are recognized as chemical markers of *U*. *guianensis* [4–6].

Natural populations of *U*. *guianensis* have declined dramatically in recent times because of strong anthropic pressure brought about mainly by deforestation and indiscriminate extraction of the bark for the commercial production of phytotherapeutic preparations [7,8]. In this context, studies on the genetic and chemical variability of medicinal plants are particularly important since they enable the selection of elite individuals that would be of interest to the pharmaceutical industry [9]. Furthermore, in the field of species conservation, molecular markers such as sequence-related amplified polymorphism (SRAP) are very useful for the identification of genetically distinct individuals with biotechnological potential [10,11]. The SRAP technique is based on five forward and six reverse primers that can be combined randomly for the amplification of a large number of open reading frames. Moreover, the SRAP method is reliable, reproducible and does not require prior knowledge of the genome [12].

Considering the ethnopharmacological and industrial importance of *U*. *guianensis*, investigations on the genetic and chemical diversity of the species would be of significant interest. Thus, the objectives of this study were to analyze the genetic diversity of natural populations of *U*. *guianensis* using SRAP markers and to determine the concentrations of mitraphylline and isomitraphylline in leaf extracts using high performance liquid chromatography (HPLC). The results of our study will contribute to the selection of individuals that could be considered elite in respect of POA concentrations and to ascertain the need for immediate conservation strategies.

## Materials and methods

### Plant material

**Fig 1:**
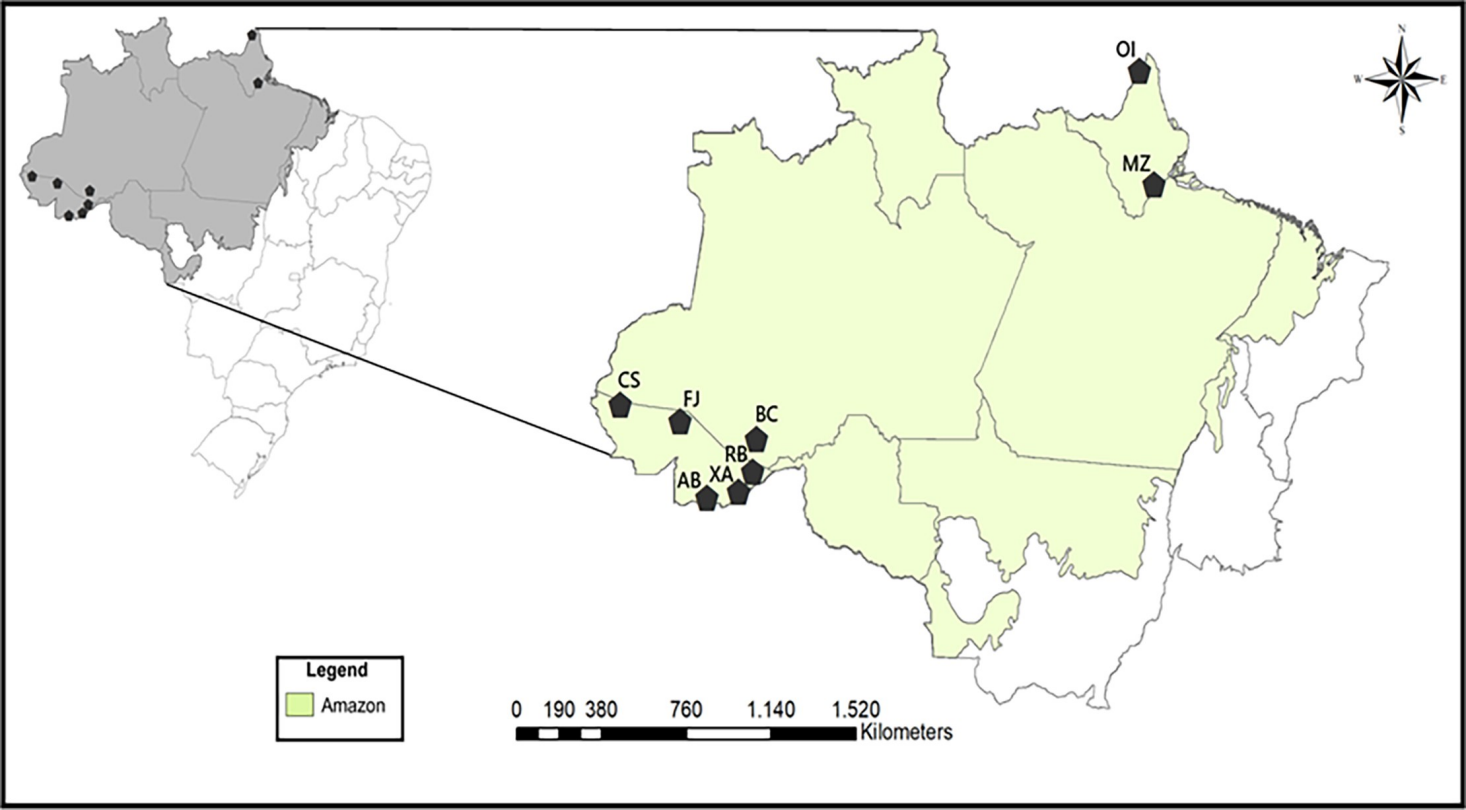
Locations in the Amazonian region of Brazil at which eight populations of *Uncaria guianensis* were sampled in this study. Key to population codes are shown in Table 1.

The collection of specimens of *U*. *guianensis* for use in the study was authorized by the Conselho de Gestão do Patrimônio Genético/Ministério do Meio Ambiente (CGEN/MMA; protocol no. 010102/2015-9) through the offices of the Conselho Nacional de Desenvolvimento Científico e Tecnológico (CNPq). A total of 157 specimens of *U*. *guianensis* were collected from eight populations located at different sites in the Amazonian region of Brazil, namely Boca do Acre in Amazonas state, Assis Brasil, Cruzeiro do Sul, Feijó, Rio Branco and Xapuri in Acre state, and Mazagão and Oiapoque in Amapá state. The identification codes of the samples are presented in Table 1 together with the geographical coordinates and altitudes of the sampling sites, while the locations of the sites are mapped in Fig 1. Specimens were identified by one us (PGD; Herbier de Guyane, Institut de Recherche pour le Développement, Cayenne, French Guiana) and voucher specimens deposited at the Herbarium of the Universidade de Ribeirão Preto (UNAERP) with identification codes HPMU2844 to 2926.

**Table 1:** Locations, geographical coordinates and altitudes of the populations of *Uncaria guianensis* sampled in this study.

Population code	Municipality, State	*N*	Latitude	Longitude	Altitude (m)	Ecology of collection site
**AB**	Assis Brasil, Acre	20	-10°48’38.7”	-69°17’48.4”	285	Adjacent to the deforested area of BR317^a^, some 344 km from the RB population. Characterized by shrubs and medium-size trees. Average distance between individuals: 70 m
**BC**	Boca do Acre, Amazonas	20	-08°47’52.0”	-67°17’21.0”	120	Anthropic area formed by secondary forest with predominance of *Cecropia* spp., small trees and shrubs. Average distance between individuals: 183 m.
**CS**	Cruzeiro do Sul, Acre	20	-07°36’44.1”	-72°48’05.7”	180	Anthropic area located 5 km from Cruzeiro do SulPlantatio, intersected by dirt roads and plantations of açaí and cassava with presence of small trees, shrubs and lianas. Average distance between individuals: 9.5 m.
**FJ**	Feijó, Acre	17	-08°11’08.1”	-70°22’26.3”	161	Anthropic area located 15 km from Feijó, with remnants of the original forest and abundance of *U*. *guianensis*. Characterized by diverse vegetation including bushes and grassland. Average distance between individuals: 110 m.
**MZ**	Mazagão, Amapá	20	+00°03’47.5”	-51°14’86.0”	12	Non-anthropic area on the bank of a narrow and shallow stream (*igarapé*) surrounded by the original forest with *U*. *guianensis*. Average distance between individuals: 2038 m
**OI**	Oiapoque, Amapá	20	+03°49’56.9”	-51°50’54.8”	4	Adjacent to BR156^b^, some 20 km from Oiapoque, surrounded by the original forest with abundance of *U*. *guianensis*. Average distance between individuals: 1490 m.
**RB**	Rio Branco, Acre	20	-09°54’44.2”	-67°26’46.8”	201	Adjacent to the deforested area of BR317^a^, with the remaining vegetation containing ruderal species interspersed with protected arboreal species such as Brazil nut trees (*Bertholletia excelsa*). Sandy soil covered by a thin layer of mulches, herbaceous plants, shrubs and various species of lianas including *U*. *guianensis*. Average distance between individuals: 1840 m
**XA**	Xapuri, Acre	20	-10°36’02.5”	-68°00’32.8”	211	Area close to Chico Mendes Extractive Reserve, some 2 km from Xapurí, characterized by large Brazil nut trees, small trees, shrubs and lianas. Average distance between individuals: 27.0 m

^a^ BR317 is a main road connecting Rio Branco (Acre) to Boca do Acre (Amazonas)

^b^ BR156 is a main road connecting Oiapoque to Macapá (Amapá)

Young healthy leaves were collected from each specimen and either stored in labeled test tubes in the freezer at -20°C until required for DNA extraction and SRAP analysis, or dried at 45°C in a forced-air oven (Marconi, Piracicaba, SP, Brazil) for HPLC analysis. All experiments were performed in the Molecular Biology and Phytochemical Laboratories of the Biotechnology Department at UNAERP.

### SRAP analysis

Genomic DNA was extracted from leaf samples (100 mg) using the cetyltrimethylammonium bromide *(*CTAB*)* method [13]. The integrity of extracted DNA was evaluated by electrophoresis on 1% agarose gels in 1 X Tris/Borate/EDTA (TBE) buffer, and quantitative evaluation was performed spectrophotometrically using a NanoDrop spectrophotometer (Thermo Fisher Scientific, Waltham, MA, USA). Samples were subsequently diluted to 5 ng/μl and submitted to SRAP analysis as described by Li and Quiros [12]. Four combinations of published forward (me) and reverse (em) primers (Table 2) that produced sharp bands with a high percentage of polymorphism were selected after testing six initial primer pairs on samples from two individuals of each population.

**Table 2:** Nucleotide sequences of the primer pairs selected for sequence-related amplified polymorphism (SRAP) analysis of *Uncaria guianensis*.

Primer pairs (forward/reverse)	Nucleotide sequence	Number of polymorphic loci	Percentage polymorphism
me1/em3	Forward: 5’-TGA GTC CAA ACC GG ATA-3’ Reverse: 5’-GAC TGC GTA CGA ATT GAC-3’	112	100
me1/em6	Forward: 5’-TGA GTC CAA ACC GG ATA-3’ Reverse: 5’-GAC TGC GTA CGA ATT GCA-3’	62	100
me3/em3	Forward: 5-TGA GTC CAA ACC GG AAT-3’ Reverse: 5’-GAC TGC GTA CGA ATT GAC-3’	30	100
me3/em1	Forward: 5-TGA GTC CAA ACC GG AAT-3’ Reverse: 5’-GAC TGC GTA CGA ATT AAT-3’	31	100
Total		235	

For all four primer pairs, polymerase chain reactions (PCR) were performed with reaction mixtures containing 1 μl of 10 X reaction buffer, 0.8 μl of MgCl_2_ (25 mM), 1 μl of dNTP mixture (2.5 mM), 0.4 μl of forward primer (5 μM), 0.4 μl of reverse primer (5 μM), 0.2 μl of Taq DNA polymerase (5 U/μl), 1 μl of DNA template (5 ng/μl) and deionized water to a final volume of 10 μl. Amplification procedures involved 5 cycles of denaturation at 94°C for 1 min, annealing at 35°C for 1 min and extension at 72°C for 1 min, followed by 35 cycles of denaturation at 94°C for 1 min, annealing at 50°C for 1 min and extension at 72°C for 1 min, with a final extension step at 72°C for 7 min.

Amplicons were denatured at 95°C for 5 min and an aliquot (0.6 μl) of each sample was applied to a KB Plus 6.50% Gel Matrix (LI-COR Biosciences, Lincoln, NE, USA) together with a 50–700 bp DNA ladder (0.8 μl). Electrophoresis was performed at 1.500 V and 40 W for 2:30 h at a constant temperature of 45°C in an LI-COR model 4300 DNA Analyzer. Primers were labeled with LI-COR 700 and 800 nm infrared dyes to allow collection of fluorescent images in real time during electrophoresis. Image data were viewed, analyzed and converted into numerical data files using LI-COR SAGA^MX^ automated analysis software version 3.3.

### Extraction and quantification of mitraphylline and isomitraphylline

A modified version of the method of Bertol et al. [14] was employed to extract mitraphylline and isomitraphylline from dried leaves of *U*. *guianensis* that had been reduced to a fine powder in a Marconi MA048 cutting mill fitted with a 40 mesh sieve. Powdered leaf material (100 mg) was mixed with 1 ml of methanol (J.T. Baker HPLC grade; Avantor Performance Materials, Center Valley, PA, USA) in an amber flask and submitted to static maceration at room temperature (22 ± 1°C) for 24 h, following which the mixture was filtered and the filtrate reduced to dryness in a fume cupboard. Triplicate extractions were performed for each of the studied specimens.

Samples (15 mg) of dried extracts were redissolved in 1 ml of an 80: 20 (*v/v*) mixture of methanol (J.T. Baker HPLC grade) and Milli-Q Ultrapure water (Merck Millipore, Darmstadt, Germany) and applied to Supelco LC-18 solid-phase extraction (SPE) tubes (Sigma, St. Louis, MO, USA) that had been previously eluted with 1 ml of methanol followed by 1 ml of 80:20 (*v/v*) methanol:water mixture. Tubes were subsequently eluted with 3 ml of 80:20 (*v/v*) methanol:water mixture and 20 μl aliquots of the eluents (5 mg/ml) were analyzed by HPLC on a Shimadzu (Kyoto, Japan) model LC*-*10ADvp instrument coupled to an SPD*-*M10Avp diode array detector (DAD). Separations were carried out at room temperature (22 ± 1°C) on a Zorbax Eclipse XDB-C18 column (150 x 4.6 mm i.d., 5 μm; Agilent, Santa Clara, CA, USA) protected by a Zorbax Eclipse XDB-C18 pre-column (4.6 x 12.5 mm i.d., 5 μm). The mobile phase comprised 10 mM aqueous ammonium acetate (pH adjusted to 6.9 with triethanolamine) (solvent A; Neon Comercial, São Paulo, Brazil) and acetonitrile (solvent B; J.T. Baker HPLC grade) and was supplied at a continuous flow rate of 0.8 ml/min according to the program: 35% B between 0.01 and 18.00 min, 50% B between 18.01 and 25.00 min, 35 to 100% B from 25.01 to 40 min, and 35% B between 40.01 and 45 min. The detection wavelength was set at 245 nm and the acquired data were processed using Shimadzu LabSolutions Multi LC-PDA software.

POA content was determined by a previously validated HPLC-DAD method [15] using mitraphylline (LGC Standards, Teddington Middlesex, UK; # CDX 00013955–005) and isomitraphylline (Chromadex, Irvine, CA, USA; # ASB-00009417-005) as external standards. Analytical data were validated with respect to linearity, precision and accuracy according to the guidelines issued by the Agência Nacional de Vigilância Sanitária [16], and limits of detection (LoD) and quantitation (LoQ) were determined to be 0.02 and 0.07 μg/ml, respectively, for mitraphylline and 0.01 and 0.02 μg/ml for isomitraphylline. Standard solutions containing POAs at concentrations of 500, 250, 125, 62.5, 31.2, 15.6, 7.8, 3.9, 1.9 μg/ml were prepared and calibration curves constructed by subjecting each solution to HPLC analysis in triplicate. The ratio of peak areas of mitraphylline and isomitraphylline standards were calculated and plotted against the corresponding standard concentrations using linear regression of the standard curves.

### Statistical analyses

The sequence data were used to perform analysis of molecular variance (AMOVA) in order to decompose the total genetic variance to within and between population components. Descriptive analysis of total variability was obtained by calculating the percentage of polymorphic loci, the observed number of alleles (N_a_), the effective number of alleles (N_e_), Nei’s genetic diversity index (H) and Shannon's diversity index (I). The unweighted pair group method with arithmetic mean (UPGMA) was used to group populations according to genetic divergence estimated from Nei’s genetic distances [17]. Geographical distances were calculated with the help of TrackMaker software version 13.8 (Geo Studio Tecnologia, Belo Horizonte, MG, Brazil).

Variability and genetic structure of populations were investigated through principal coordinate analysis (PCoA) using the software packages GenAlEx version 6.5 [18] and STRUCTURE version 2.2.4 [19,20]. The most likely number of population groups was established using the Bayesian model-based clustering algorithm (in which individuals are assigned to K population genetic clusters based on their nuclear multilocus genotypes) and the admixed ancestry model. For each run, the initial burn-in was 200,000 iterations followed by a run-length of 500,000 iterations for K = 1 to 10 population genetic clusters.

Data relating to the accumulation of mitraphylline and isomitraphylline within and between populations were submitted to analysis of variance (ANOVA) and, when significant differences were detected, mean values were compared using the Scott-Knott test at 5% probability. A dendrogram was constructed using the UPGMA clustering method to establish the organization of chemical variables among the evaluated populations. The matrix-based cophenetic correlations produced by the UPGMA dendrogram were calculated using the *vegan* and *ecodist* R packages [21]. The Euclidian distance matrix of chemical data (POA content) was correlated with geographical distance, altitude and Nei’s genetic distance matrices. Pairwise relationships between populations were evaluated using simple Mantel tests with 10,000 permutations performed with the aid of *vegan*, *fields* and *ecodist* R packages.

## Results and discussion

### Genetic diversity within and between *U*. *guianensis* populations

Of the four primer pairs studied, me1/em3 generated the highest number of bands, while me3/me3 produced the smallest number, yielding 235 amplified bands with 100% polymorphism (Table 2). The highest percentage variability (90.21%) was observed within the Mazagão population (MZ), which was collected from an *igarapé* (a narrow, shallow tributary) located in a non-anthropized area (Table 3 and S1 Table). In contrast, the lowest percentage variability (39.57%) was observed within the Assis Brazil population (AB), which is located on the margins of the BR317 highway (Table 3 and S1 Table)

**Table 3:** Genetic parameters of the populations of *Uncaria guianensis* collected in the Amazonian region of Brazil.

Population code ^a^	Percentage polymorphic loci	Observed number of alleles ^b^ (N_a_)	Expected number of alleles (N_e_)	Nei's index (H)	Shannon's index (I)
**AB**	39.57	1.39	1.16	0.1000	0.1596
**BC**	76.60	1.76	1.23	0.1582	0.2617
**CS**	73.62	1.73	1.35	0.2103	0.3237
**FJ**	67.23	1.67	1.36	0.2080	0.3138
**MZ**	90.21	1.90	1.45	0.2846	0.4376
**OI**	85.96	1.85	1.38	0.2414	0.3776
**RB**	84.68	1.84	1.28	0.1875	0.3058
**XA**	56.17	1.56	1.23	0.1416	0.2214
**Total**	98.30	1.98	1.35	0.2289	0.3686

^a^ Population codes are defined in Table 1.

^b^ Mean values

Mantel tests revealed a positive but not significant (r = 0.33; *P* > 0.0308) correlation between geographical and genetic distances among the eight *U*. *guianensis* populations (Table 4), indicating that the geographical distribution of the populations did not influence the organization of their genetic variability. In general, geographic distribution and evolutionary history play important roles in the genetic structure of a population [22].

**Table 4:** Geographical and genetic distances between populations of *Uncaria guianensis* collected in the Amazonian region of Brazil.

	RB	BC	MZ	OI	CS	FJ	XA	AB
**RB**		111	2088	2274	644	359	112	242
**BC**	0.0191		2018	2183	623	349	217	316
**MZ**	0.0493	0.0460		398	2534	2296	2186	2341
**OI**	0.0784	0.0558	0.0375		2624	2409	2353	2487
**CS**	0.0519	0.0475	0.0478	0.0455		279	622	525
**FJ**	0.0441	0.0452	0.0514	0.0519	0.0123		378	321
**XA**	0.0693	0.0640	0.0760	0.0713	0.0354	0.0460		146
**AB**	0.0888	0.0732	0.0818	0.0730	0.0442	0.0636	0.0187	

Geographic distances (km) are shown above the diagonal line while genetic distances are shown below the line.

Population codes are defined in Table 1.

The results from AMOVA (Table 5) revealed that 19% of the observed genetic variability was associated with the between population component, as shown by the F_ST_ value of 0.188 (*P* > 0.001). On this basis, the conservation of *U*. *tomentosa* should prioritize the collection of as many individuals as possible within populations that presented the highest genetic variability. For allogamous species, the within population genetic variability is expected to be high [23], hence it is likely that *U*. *guianensis* is allogenic, although the mechanism of fertilization of this species has yet to be described. It is important to emphasize that during the collection of specimens of *U*. *guianensis* for the present study, we observed extensive areas of fragmented forest, a situation that will certainly lead to the diminution of species diversity in the medium term.

**Table 5:** Genetic variability within and between populations of *Uncaria guianensis* collected in the Amazonian region of Brazil.

Source	Degrees of freedom	Mean square error	Variance components	Percentage variability	*P*	Fixation index F_ST_
**Within populations**	149	25.747	25.747	81		
**Between populations**	7	143.047	5.980	19	> 0.001	0.188
**Total**	156		31.728	100		

Data estimated by analysis of molecular variance (AMOVA).

The genetic differentiation between populations of *U*. *guianensis* (F_st_ = 0.188) was high but slightly lower than that observed for *U*. *tomentosa* (F_st_ = 0.246) [8]. However, this index can be very variable among species of the same family and/or genus that inhabit a common biome, as has been described for populations of the Amazonian medicinal plant *Psychotria ipecacuanha* (Rubiaceae), which presented high F_st_ values ranging from 0.355 to 0.457 [24].

**Fig 2:**
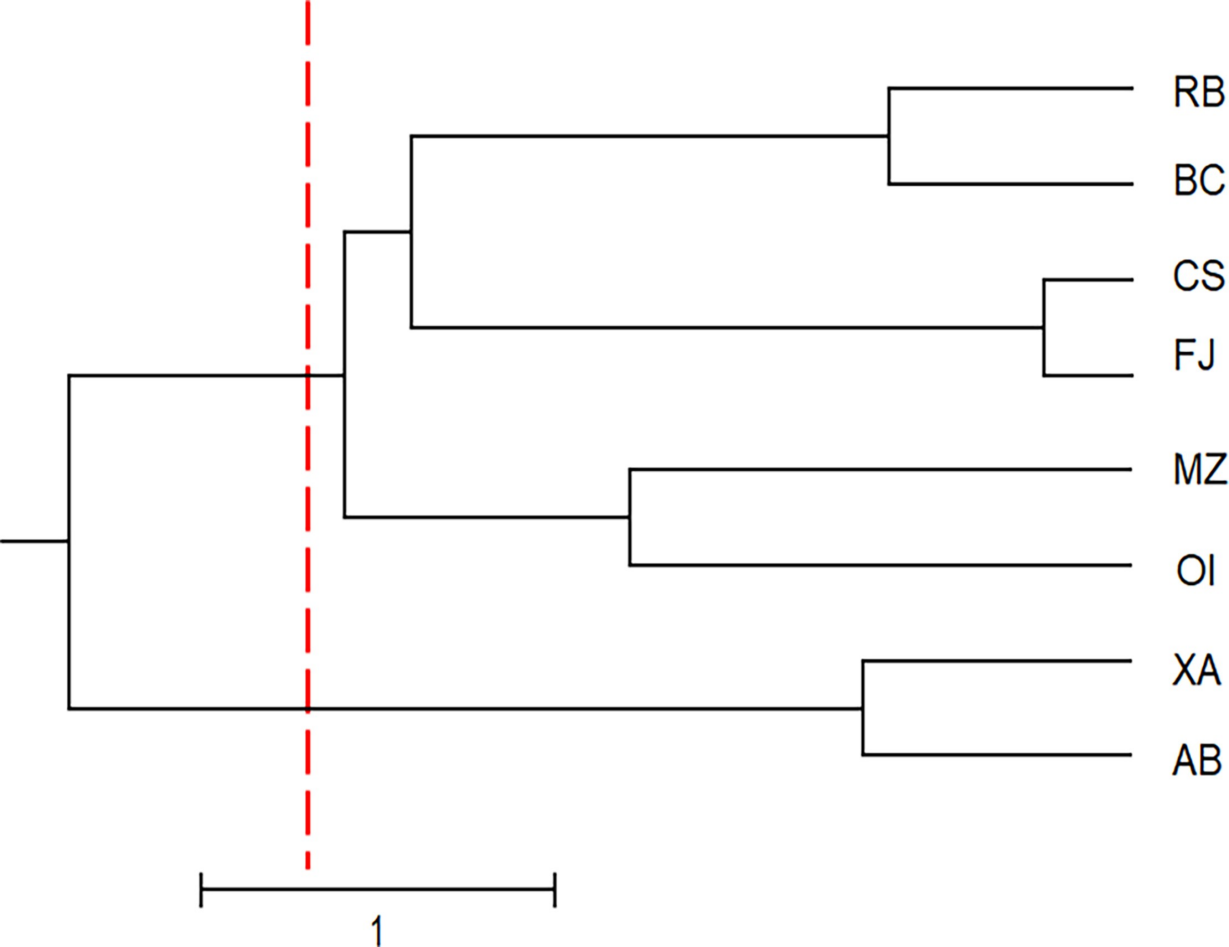
Dendrogram showing the classification of the studied populations of *Uncaria guianensis* into three genetic groups.

**Fig 3:**
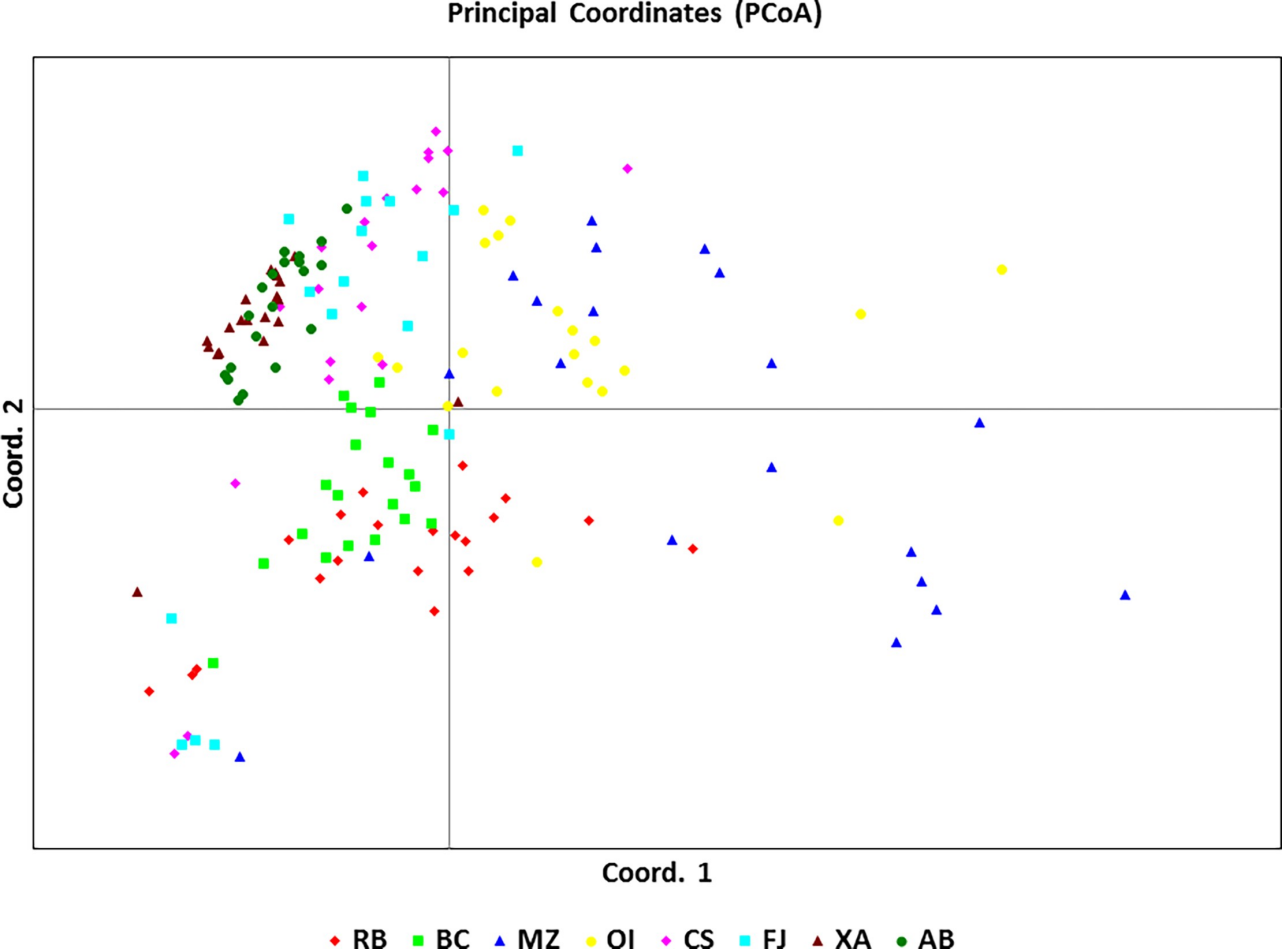
Analysis of the dispersion of the principal coordinates obtained from the Jaccard similarity matrix created with SRAP molecular markers among individuals from eight natural populations of *Uncaria guianensis*. 3.

**Fig 4:**
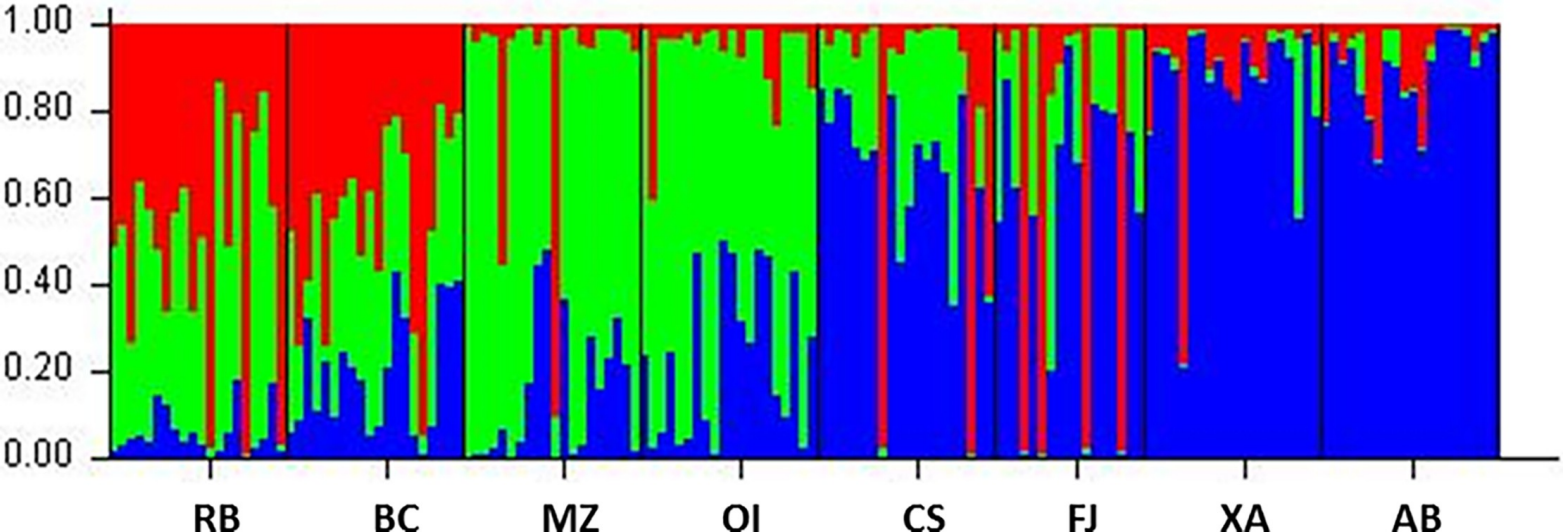
Bayesian analysis, performed using STRUCTURE software, of eight populations of *Uncaria guianensis* from the Amazon region of Brazil showing the tendency to form three clusters. Group 1 (predominantly red): Rio Branco (RB) and Boca do Acre (BC); Group 2 (predominantly green): Mazagão (MZ) and Oiapoque (OI); Group 3 (predominantly blue): Cruzeiro do Sul (CS), Feijó (FJ), Xapuri (XA) and Assis Brasil (AB).

The UPGMA dendrogram (Fig 2), the PCoA plot (Fig 3) and Bayesian analysis performed using STRUCTURE software (Fig 4) revealed that the eight populations of *U*. *guianensis* tended to form three genetic clusters (K = 3). The formation of three genetic clusters clearly demonstrates that the environment was fragmented by human interference, rendering the species vulnerable to genetic erosion and indicating the need to implement conservation strategies.

### Variation of POA content within and between *U*. *guianensis* populations

Although mitraphylline and isomitraphylline are considered to be chemical markers of *U*. *guianensis*, the concentrations of these POAs varied considerably within the populations studied (S1 Fig). Some individuals (7.6%) accumulated only isomitraphylline, others (11.5%) accumulated only mitraphylline, a few (12.7%) accumulated both alkaloids, while most (68.1%) accumulated neither, as for example the members of the MZ and OI populations (Table 6). The AB population accumulated the highest amounts of mitraphylline (0.60 mg/g dw) of all eight populations studied (Table 7). Interestingly, one individual (no. 17) from population BC presented five times more mitraphylline (2.69 mg/g dw) than the average of all other mitraphylline-producing individuals (0.5 mg/g dw). The discovery of POA-producing elite specimens is important because such individuals can become targets for large scale multiplication and conservation with the aim of providing quality raw material for the pharmaceutical industry.

**Table 6:** Mean concentrations of mitraphylline (Mit) and isomitraphylline (Iso) in populations of *Uncaria guianensis* collected in the Amazonian region of Brazil.

Population ^a^ / alkaloid		Concentrations of pentacyclic oxindole alkaloids in individual specimens (mg/g dw)																			
		1	2	3	4	5	6	7	8	9	10	11	12	13	14	15	16	17	18	19	20
**RB**	**Mit**	0.22a	0.00b	0.00b	0.00b	0.00b	0.00b	0.00b	0.00b	0.00b	0.00b	0.00b	0.00b	0.00b	0.00b	0.00b	0.19a	0.00b	0.00b	0.00b	0.00b
	**Iso**	0.29a	0.00f	0.08d	0.00f	0.00f	0.00f	0.00f	0.00f	0.00f	0.00f	0.00f	0.00f	0.00f	0.16b	0.27a	0.13c	0.18b	0.04e	0.00f	0.06d
**BC**	**Mit**	0.00c	0.00c	0.00c	0.00c	0.00c	0.00c	0.00c	0.00c	0.00c	0.00c	0.00c	0.00c	0.00c	0.00c	0.98b	0.96b	2.69a	0.00c	0.00c	0.30c
	**Iso**	0.00c	0.00c	0.00c	0.00c	0.00c	0.00c	0.00c	0.00c	0.00c	0.00c	0.00c	0.00c	0.00c	0.00c	0.07c	0.16c	0.68a	0.21c	0.49b	0.08c
**OI**	**Mit**	0.00a	0.00a	0.00a	0.00a	0.00a	0.00a	0.00a	0.00a	0.00a	0.00a	0.00a	0.00a	0.00a	0.00a	0.00a	0.00a	0.00a	0.00a	0.00a	0.00a
	**Iso**	0.00a	0.00a	0.00a	0.00a	0.00a	0.00a	0.00a	0.00a	0.00a	0.00a	0.00a	0.00a	0.00a	0.00a	0.00a	0.00a	0.00a	0.00a	0.00a	0.00a
**MZ**	**Mit**	0.00a	0.00a	0.00a	0.00a	0.00a	0.00a	0.00a	0.00a	0.00a	0.00a	0.00a	0.00a	0.00a	0.00a	0.00a	0.00a	0.00a	0.00a	0.00a	0.00a
	**Iso**	0.00a	0.00a	0.00a	0.00a	0.00a	0.00a	0.00a	0.00a	0.00a	0.00a	0.00a	0.00a	0.00a	0.00a	0.00a	0.00a	0.00a	0.00a	0.00a	0.00a
**CS**	**Mit**	0.00a	0.00a	0.00a	0.00a	0.00a	0.00a	0.00a	0.00a	0.00a	0.00a	0.00a	0.00a	0.00a	0.00a	0.00a	0.00a	0.00a	0.00a	0.00a	0.00a
	**Iso**	0.00c	0.00c	0.00c	0.05b	0.11a	0.02c	0.00c	0.00c	0.00c	0.00c	0.00c	0.00c	0.00c	0.00c	0.00c	0.00c	0.00c	0.00c	0.00c	0.00c
**FJ**	**Mit**	0.00b	0.00b	0.00b	0.00b	0.00b	0.00b	0.00b	0.00b	0.00b	0.00b	0.00b	0.00b	0.00b	0.00b	0.00b	0.49a	0.00b	*	*	*
	**Iso**	0.00b	0.00b	0.00b	0.00b	0.00b	0.00b	0.00b	0.00b	0.00b	0.00b	0.00b	0.00b	0.00b	0.00b	0.00b	0.10a	0.03b	*	*	*
**XA**	**Mit**	0.00g	0.00g	0.00g	0.00g	0.60c	0.89b	0.00g	0.04f	0.27d	0.00g	0.05f	0.13e	0.01g	0.04f	0.01g	0.04f	0.00g	1.24a	0.00g	0.08f
	**Iso**	0.00c	0.00c	0.00c	0.00c	0.04b	0.52a	0.00c	0.00c	0.00c	0.00c	0.00c	0.00c	0.00c	0.00c	0.02b	0.00c	0.00c	0.02b	0.00c	0.00c
**AB**	**Mit**	0.79c	0.39e	0.92c	1.00b	0.29e	1.17a	0.47d	0.56d	0.78c	0.52d	0.63d	0.57d	0.13f	0.55d	0.51d	0.44d	0.38e	0.69d	0.54d	0.58d
	**Iso**	0.37a	0.00e	0.02e	0.25c	0.01e	0.30b	0.09d	0.01e	0.00e	0.09d	0.00e	0.00e	0.00e	0.00e	0.00e	0.00e	0.00e	0.00e	0.00e	0.02e

In each row, mean values bearing dissimilar letters are significantly different according to Scott-Knott test at 5% probability.

* not sampled.

^a^ Population codes are defined in Table 1.

**Table 7:** Mean concentrations of mitraphylline and isomitraphylline in populations of *Uncaria guianensis* collected in the Amazonian region of Brazil.

Population code ^a^	Mitraphylline (mg/g dw)	Isomitraphylline (mg/g dw)
**AB**	0.60a	0.06a
**BC**	0.25b	0.09a
**CS**	0.00c	0.01a
**FJ**	0.03c	0.03a
**MZ**	0.00c	0.00a
**OI**	0.00c	0.00a
**RB**	0.02c	0.06a
**XA**	0.17b	0.03a

In each column, mean values bearing dissimilar letters are significantly different according to Scott-Knott test at 5% probability.

^a^ Population codes are defined in Table 1

The OI and MZ populations exhibited genetic and chemical similarity, and this profile may be related to the low altitude of these two populations (4–12 m). An association between altitude and POA content has also been observed for *U*. *tomentosa* in which the accumulation of these alkaloids was maximal in plants growing at altitudes above 230 m [15].

## Conclusions

Analyses employing polymorphic SRAP markers enabled the genetic variability within and between eight natural populations of *U*. *guianensis* to be characterized. It was possible to detect the presence of three genetic groups, indicating the need to develop conservation strategies in order to preserve such variability. Geographic distance did not explain the distribution of genetic variability between the studied populations. Since genetic variability was higher within populations, we suggest that the best conservation strategy would involve the collection of large numbers of individuals from populations presenting the highest variability. Although a few POA-rich individuals were detected, the majority of specimens did not accumulate mitraphylline and/or isomitraphylline. This finding suggests that these two POAs are not the best chemical markers for *U*. *guianensis* and that further studies are required in order to understand the heritability of chemical traits in this species for domestication and breeding purposes. The scarceness of POA-rich specimens of *U*. *guianensis* means that commercial production of phytotherapeutics based on this species may be problematic since it depends on the collection of specimens from natural environments without previous selection. We conclude that there is an urgent need for conservation projects involving *U*. *guianensis*, with particular emphasis on the creation of germplasm banks.

## Supporting information

S1 Fig High performance liquid chromatographic (HPLC) analyses of leaf extracts of three specimens of *Uncaria guianensis* populations from the Amazonian region of Brazil. The chromatograms show the standards (**A**) mitraphylline and (**B**) isomitraphylline, along with extracts from specimens collected in (**C**) Boca do Acre, AM (BC) in which peaks labeled **a** correspond to mitraphylline, (**D**) Assis Brasil, AC (AB) in which the peak labeled **b** corresponds to isomitraphylline, (**E**) Xapuri, AC (XA) in which peaks labeled **a** and **b** correspond to mitraphylline and isomitraphylline, respectively, and (**F**) Rio Branco, AC (RB) in which peaks **a** and **b** are absent.
(DOCX)

S1 Table Population parameters for all four primer pairs employed in SRAP analysis of *Uncaria guianensis* populations from the Amazonian region of Brazil. (XLSX)
