# Supplementary material for: Genetic structure and chemical diversity in natural populations of Uncaria guianensis (Aubl.) J.F.Gmel. (Rubiaceae)
Source: PLoS One. 2018 Oct 26;13(10):e0205667. doi: 10.1371/journal.pone.0205667 (PMC6203251; doi:10.1371/journal.pone.0205667)
Supplement: S1 Fig — The chromatograms show the standards (A) mitraphylline and (B) isomitraphylline, along with extracts from specimens collected in (C) Boca do Acre, AM (BC) in which peaks labeled a correspond to mitraphylline, (D) Assis Brasil, AC (AB) in which the peak labeled b corresponds to isomitraphylline, (E) Xapuri, AC (XA) in which peaks labeled a and b correspond to mitraphylline and isomitraphylline, respectively, and (F) Rio Branco, AC (RB) in which peaks a and b are absent. (DOCX) [file pone.0205667.s001.docx]

**Supporting Information**

**Genetic structure and chemical diversity**

**in natural populations of**

***Uncaria guianensis* (Aubl.) J.F.Gmel. (Rubiaceae)**

Isabela C. G. Honório^1^, Juliana S. Coppede^2^, Piero G. Delprete^3^, Frederico H. S. Costa^4^, Mariana P. C. Telles^5^, Ramilla S. Braga^6^, José A. F. Diniz-Filho^7^, Suzeli C. França^2^, Ana M. S. Pereira^2*^, Bianca W. Bertoni^2^

^1^ Universidade Estadual Paulista “Júlio de Mesquita Filho”, Campus Lageado, Botucatu, SP, Brazil

^2^ Universidade de Ribeirão Preto, Ribeirânia, Ribeirão Preto, SP, Brazil

^3^ Herbier de Guyane, Institut de Recherche pour le Développement, Cayenne, French Guiana

^4^ Escola de Ciências Agrárias e Biológicas, Pontifícia Universidade Católica de Goiás, Goiânia, GO, Brazil

^4^ Laboratório de Genética e Biodiversidade, Universidade Federal de Goiás, Campus Samambaia, Goiânia, GO, Brazil

^5^ Laboratório de Genética e Biodiversidade, Universidade Federal de Goiás, Campus Samambaia, Goiânia, GO, Brazil

^6^ Reserva EcoCerrado Brasil, Araxá, MG, Brazil

^7^ Departamento de Ecologia, ICB, Universidade Federal de Goiás, Goiânia, GO, Brazil

* Corresponding author

E-mail: [apereira@unaerp.br](mailto:apereira@unaerp.br) .

**Figure S1. High performance liquid chromatographic (HPLC) analyses of leaf extracts of three specimens of *Uncaria guianensis* populations from the Amazonian region of Brazil.** The chromatograms show the standards (**A**) mitraphylline and (**B**) isomitraphylline, along with extracts from specimens collected in (**C**) Boca do Acre, AM (BC) in which peaks labeled **a** correspond to mitraphylline, (**D**) Assis Brasil, AC (AB) in which the peak labeled **b** corresponds to isomitraphylline, (**E**) Xapuri, AC (XA) in which peaks labeled **a** and **b** correspond to mitraphylline and isomitraphylline, respectively, and (**F**) Rio Branco, AC (RB) in which peaks **a** and **b** are absent.

**Table S1. Population parameters for all four primer pairs employed in SRAP analysis of *Uncaria guianensis* populations from the Amazonian region of Brazil.**

(XLSX)

b

a

a

C

B

A

D

b

E

a

b

F

F
